# Rigid registration algorithm based on the minimization of the total variation of the difference map

**DOI:** 10.1107/S1600577522005598

**Published:** 2022-06-20

**Authors:** Xianghui Xiao, Zhengrui Xu, Dong Hou, Zhijie Yang, Feng Lin

**Affiliations:** a National Synchrotron Light Source II, Brookhaven National Laboratory, Upton, NY 11973, USA; b Department of Chemistry, Virginia Tech, Blacksburg, VA 24061, USA

**Keywords:** difference map, image registration, total variation, X-ray microscopy, TXM-XANES

## Abstract

Image registration is broadly used in various scenarios in which similar scenes in different images are to be aligned. However, image registration becomes challenging when the contrasts and backgrounds in the images are vastly different. This work proposes using the total variation of the difference map between two images (TVDM) as a dissimilarity metric in rigid registration. A method based on TVDM minimization is implemented for image rigid registration. The method is tested with both synthesized and real experimental data that have various noise and background conditions. The performance of the proposed method is compared with the results of other rigid registration methods. It is demonstrated that the proposed method is highly accurate and robust and outperforms other methods in all of the tests. The new algorithm provides a robust option for image registrations that are critical to many nano-scale X-ray imaging and microscopy applications.

## Abstract 2

A new dissimilarity metric based on the total variation of the difference map between an image pair is proposed for rigid registration applications. A rigid registration method based on a new metric – total variation of the difference map – is tested and compared with the Fourier analysis-based phase correlation and sum of squared difference metrics.

## 1. Introduction

Image registration is a process of mapping related images of the same object taken at different times, or different angles, or under different environmental conditions, or with different sensors to integrate information about the features of interest. Image registration has broad applications in computer vision, remote sensing (Tondewad & Dale, 2020 ▸; Dawn *et al.*, 2010 ▸), and medical imaging (Maintz & Viergever, 1998 ▸; Sotiras *et al.*, 2013 ▸) tasks, among many others. In the data analysis in nano-scale X-ray imaging and microscopy, *e.g.* transmission X-ray microscopy based XANES (TXM-XANES), image registration is a critical data pre-processing step (Xiao *et al.*, 2022 ▸; Yu *et al.*, 2018 ▸; Lerotic *et al.*, 2014 ▸). In such applications, the features’ contrasts and intensities, background patterns and noise levels in the images may vary significantly. These variations make precise image registration a challenging task. On the other hand, image registration of series of images with sub-pixel level precision is crucial for nano-scale imaging data analysis like TXM-XANES. Although many off-the-shelf image registration algorithms have been applied for such tasks, light-weight robust algorithms that can work reliably in these non-ideal and diverse scenarios are still lacking.

In general, the registration between a target image *I*_t_ and a source image *I*_s_ can be formulated as an optimization problem in which the dissimilarity between *I*_t_ and some form of transformation of *I*_s_ is minimized. The goal of the registration operation is to have the transformed *I*_s_ match *I*_t_ as close as possible.

Total variation (TV) regulation (Rudin *et al.*, 1992 ▸) has long been used in ill-posed deformable image registration (Henn, 2003 ▸; Hömke *et al.*, 2007 ▸),

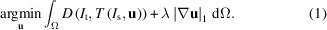




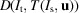
 is a function to quantify the dissimilarity between *I*_t_ and the transformed image 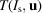 : 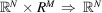 , **u** ∈ *R* ^*M*^ is the displacement field, 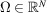 is the image space, *N* is the number of pixels of the images, *M* is the dimensionality of the images, |∇**u**|_1_ is the TV of **u** defined as the L1 norm of its gradient, and λ is a relaxation factor. The TV regulation helps to constrain the optimization problem and reduce the outliers but preserve the discontinuity in the calculated displacement field. For simplicity, we will only discuss two-dimensional image registration in the following sections, but all the conclusions can be easily extended to three-dimensions cases.

Image rigid registration is a type of registration widely used in many applications, including brain and orthopedic imaging (Hill *et al.*, 2001 ▸; El-Gamal *et al.*, 2016 ▸). In rigid-body transformation, the freedoms are reduced to only global translation and rotation. In the rigid-body case, the displacement field **u**(*x*) can be expressed as (Eggert *et al.*, 1997 ▸)

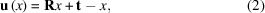

where *x* ∈ *R* ^*M*^ is the position of a pixel in the target image, **R** is the rotation transform and **t** is a translation vector. **R** *x* + **t** is the position of the corresponding pixel in the source image. Therefore, **u**(*x*) defines the positional displacement map between the corresponding pixels in target and source images. In a 2D case, it is easy to verify that |∇**u**|_1_ in equation (1) is 2*A*(1 − cosα + |sinα|), *A* is the area of the image space Ω, and α is the relative rotation angle between two images. That is a quantity independent of the translation **t** and thus does not enforce right constraint in a general rigid-body image registration task. Therefore, TV regulation on the displacement field is not as efficient as that in deformable registration applications. Mathematically, some form of regulation is not necessary in ideal rigid registration (Zitová & Flusser, 2003 ▸) since there are only three and six unknown transform parameters to be determined in 2D and 3D cases, respectively. The problem is not intrinsically underdetermined if noise is not concerned. Therefore, the rigid-body registration methods are designed to determine the transform parameters based on either the selected point sets or the intensities of two images (Zitová & Flusser, 2003 ▸; Tang & Hamarneh, 2013 ▸). Compared with the point cloud-based methods, the intensity-based methods save the point set detection step. The intensity-based methods are more suitable in general applications because there is not a universal way to select corresponding point sets from the images that have different contrasts and artifacts. Nonetheless, strong noise, irregular backgrounds or artifacts from image resampling may still affect the performances of the intensity-based rigid-body registration methods (Tang & Hamarneh, 2013 ▸).

In this work, we propose a new dissimilarity metric based on the total variation of the difference map between a target image and a source image. It is demonstrated that the new metrics can effectively suppress the effects of variable and noisy backgrounds and thus provides more robust performance compared with other intensity-based rigid-body registration methods.

## 2. Image registration with total variation as a dissimilarity metrics

**Figure 1:**
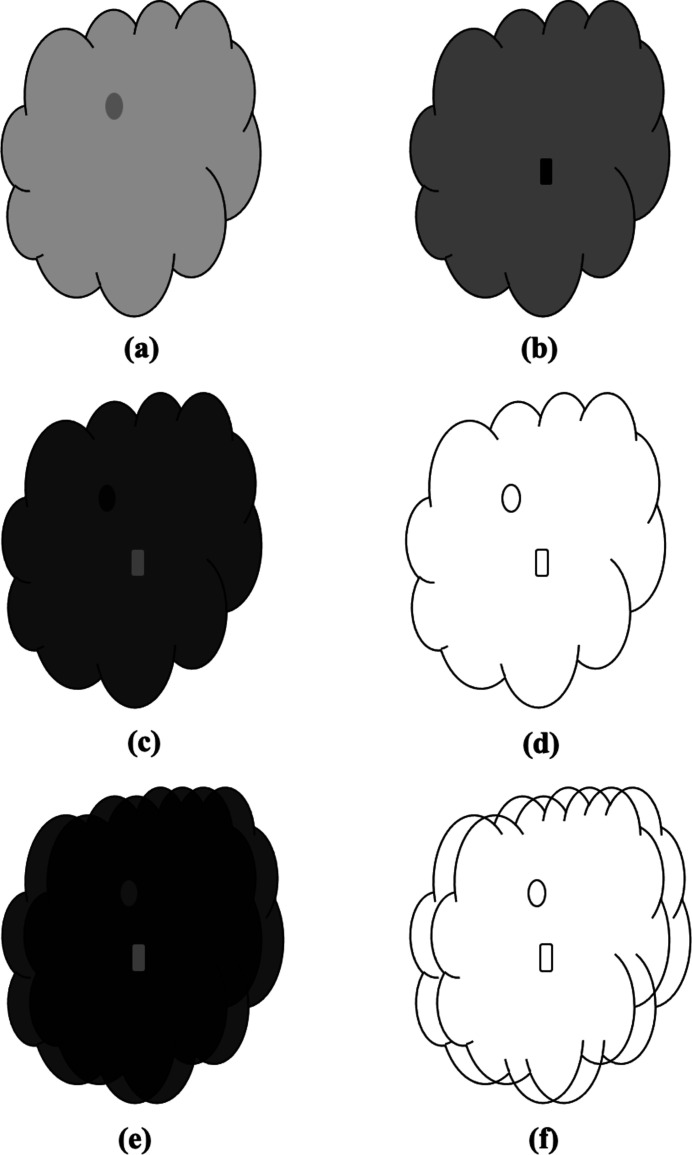
Schematic illustration of the proposed TVDM principle for aligning similar images. Panels (*a*) and (*b*) show the target and source images *I*_t_ and *I*_s_. (*c*) *I*_t_ − *I*_s_ when two images are aligned. It has non-zero background since the gray scales of *I*_t_ and *I*_s_ are not calibrated. (*d*) Gradient map of (*c*). The non-zero background is removed from the gradient operation, so only edges are reserved. (*e*) *I*_t_ − *I*_s_ when two images are not aligned. (*f*) Gradient map of (*e*). The number of edge points is doubled compared with that in (*d*).

A difference map between two images *I*_t_ and *I*_s_ is defined as *d*_m_ = *I*_s_ − *I*_t_. Difference maps are usually used to highlight differences between two images. However, as illustrated in Fig. 1 ▸, the difference map 1(*c*) of the image pair 1(*a*) and 1(*b*) renders the edge features on top of a non-zero background if 1(*a*) and 1(*b*) have different intensity scales. The L1 or L2 norm of a difference map like Fig. 1 ▸(*c*) might be dominated by the non-zero background and thus may not be sensitive to the misaligned features that are usually in the edge regions. To reduce the effects of the non-zero backgrounds in the difference map, a gray value scale factor between two images is fitted as one unknown parameter in the registration process (Ashburner & Friston, 2007 ▸). Nonetheless, a global scaling factor may not remove the non-zero background effects if two images have different contrasts and backgrounds (Tang & Hamarneh, 2013 ▸). This is the case when two images are acquired with different sensors or under different conditions.

The gradient of the difference map ∇*d*_m_ can effectively remove the non-zero background. As illustrated in Fig. 1 ▸(*d*), the gradient of Fig. 1 ▸(*c*) has significant values only around the edges. When two images are not aligned, there would be more non-zero areas presented in the different map, as illustrated in Fig. 1 ▸(*e*). Nonetheless, the gradient operation on the difference map still removes the non-zero background and highlights the residual edges. In such cases, as suggested by Fig. 1 ▸(*f*), the number of pixels with significant values is doubled compared with that when two images are aligned [Fig. 1 ▸(*c*)]. Heuristically, ∇*d*_m_ can be used as a measure of the alignment between two images.

This leads to using the total variation of the difference map between the registered image pair as a cost function in image registration,

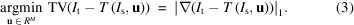

Compared with sum of squared difference and cross correlation, which are two widely used dissimilarity metrics, the total variation of the difference map (TVDM) weights more on the features in the discontinuous regions that are more distinguished features when two images are not perfectly aligned. It is also less sensitive to the non-uniform but smoothly variant background features in the image pair. Such smooth background features are largely suppressed after the gradient operation.

It should be noticed that the proposed method in this work is different from the methods that use the total variation of the displacement field as a regulation term. The displacement field as defined in equation (2) is the pixel coordinate displacement map between all corresponding pixels in the target and source images. On the other hand, the difference map used in TVDM is the direct subtraction between two images. TVDM is used as a sole cost function rather than a regulation term in the image registration. In Section 4, we will show the difference between the proposed method and a method using total variation of the displacement field as a regulation term in the scope of rigid registration.

## 3. Implementation of TVDM based rigid-body registration

**Figure 2:**
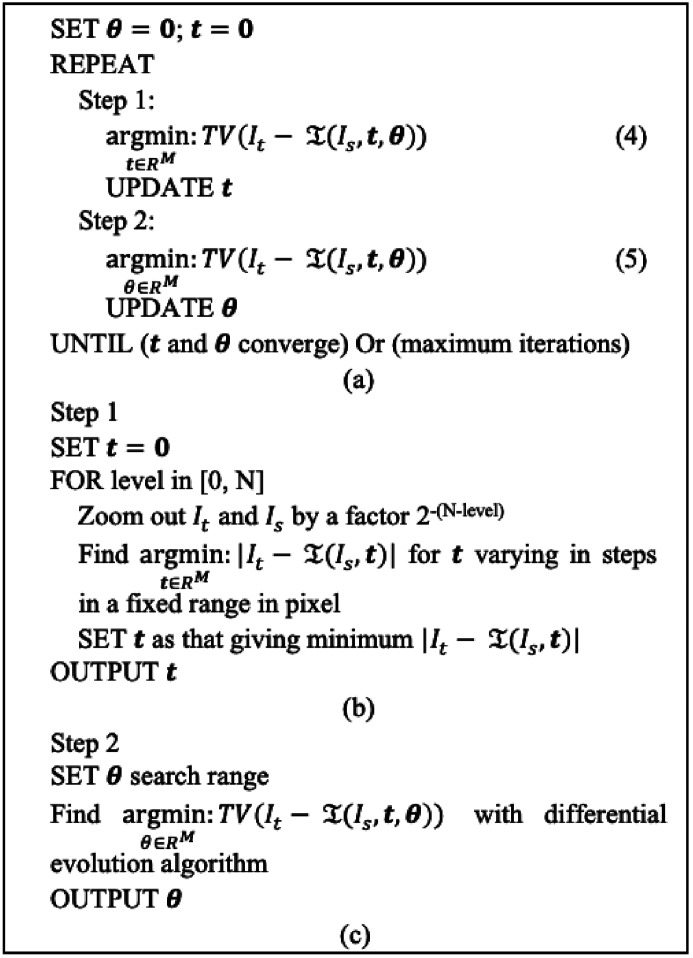
(*a*) Pseudocode for the minimization of equation (3) in 2D rigid-body registration. (*b*) Pseudocode for Step 1 in (*a*). (*c*) Pseudocode for Step 2 in (*a*).

In rigid-body cases, the transform function 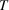 in equation (3) transforms a pixel at *x* in the source image *I*_s_ to a new position **R** *x* + **t**, according to equation (2). As in other rigid-body registration approaches, the search of matched rotation angles and translation can be conducted separately in sequence as in other rigid registration methods (Huang *et al.*, 1986 ▸; Arun *et al.*, 1987 ▸). The pseudocode for solving the optimization problem in equation (3) is shown in Fig. 2 ▸.

There is not a unique solution if the feature pattern in the images poses certain symmetries. Two example illustrations of such special symmetries are given in the supporting information. Note that there is not a specific issue with the proposed TVDM method. For instance, the cost function based on the sum of squared difference cannot distinguish the degenerate orientations of an object with a central symmetry either. In a general case, the feature patterns in images are usually complicated, so rarely have such special symmetries. Therefore, the proposed method should work in general cases like methods using other types of similarity metrics but with better robustness and higher sensitivities to the misalignment features, which will be demonstrated with examples in the next section.

Different minimization schemes are employed in the minimizations of equations (4) ▸ and (5) ▸ in Fig. 2 ▸(*a*) to accommodate the difference between them. For equation (4) ▸, a multi-resolution minimization is adopted in which a brute-force search in a small region is applied at each resolution level, with the result from the lower-resolution level as the center of the search range in the next higher-resolution level. This search usually leads to a state where two images are partially overlapped which is good for the angle search in the next step. The pseudocode of this procedure is provided in Fig. 2 ▸(*b*). For equation (5) ▸, a stochastic optimization algorithm – the differential evolution (DE) (Storn & Price, 1997 ▸) – is employed to find the global minimum. The DE algorithm iteratively searches the rotation parameter space by mutating and selecting between a population of candidate solutions to reach an optimal point. DE does not assume that the optimization problem is differentiable so is suitable for the problem defined in equation (5) ▸. DE is demonstrated to be superior to many other optimization algorithms in terms of the required number of function evaluations necessary to locate a global minimum (Storn & Price, 1997 ▸; Das & Suganthan, 2011 ▸; Das *et al.*, 2016 ▸). In this work, we used the DE implementation available in the *Scipy.optimize* package (Virtanen *et al.*, 2020 ▸). The pseudocode for the minimization of equation (5) ▸ is presented in Fig. 2 ▸(*c*). Since the translation and rotation angle correction are coupled, the minimizations of equations (4) and (5) ▸ are iterated until a stable result or the maximum number of iterations is reached.

In practice, there is always noise in images that can decorate the edge features. A Gaussian filter on the images can effectively reduce the noise effect in TVDM. Therefore, *I*_t_ and *I*_s_ in the pseudocode above are replaced with their Gaussian filtered version gf(*I*_t_, σ)gf(*I*_s_, σ), where σ is the standard deviation for the filter. It is found that σ = 1 is efficient in most cases.

## 4. Experiments

For simplicity, we only focus on 2D examples in this work to demonstrate the validity of using TVDM as an optimization metric in rigid-body registration. The proposed method is tested with both the simulated and experimental data, and its performance is compared with three other algorithms. The popular dissimilarity metrics used in image registrations include, for example, the sum of squared difference, the normalized cross correlation, the phase correlation, and the mutual information (Zitová & Flusser, 2003 ▸), to name a few. Thorough discussions on the dissimilarity metric topic can be found in review articles (Song *et al.*, 2017 ▸; Wyawahare *et al.*, 2009 ▸; Zitová & Flusser, 2003 ▸, and references therein). For comparisons with the proposed method, we choose one method based on phase correlation, one based on the sum of squared difference, and one method using the total variation of the displacement field as the regulation term for the minimization in the registration. For the phase correlation method, we use a Python implementation available at https://github.com/YoshiRi/ImRegPOC (Ri & Fujimoto, 2018 ▸) and refer to it as FPC. This algorithm implements a band filter to reduce both low-frequency background and high-frequency noise. For the sum of squared difference based method, a routine implemented in the popular image processing package *Insight Toolkit* (McCormick *et al.*, 2014 ▸) via the SimpleElastix (Marstal *et al.*, 2016 ▸) interface to Python is used. The ‘AdvancedMeanSquares’ metric and ‘StandardGradientDescent’ optimizer are used in this routine. It is referred to as the SSD method in this work. For the third method, the routine optical_flow_tvl1 (Zach *et al.*, 2007 ▸; Wedel *et al.*, 2009 ▸; Sánchez Pérez *et al.*, 2013 ▸) available in *scikit-image*’s registration module (van der Walt *et al.*, 2014 ▸) is used. This is a deformable registration method that uses the L1-norm of the difference image as the cost function and the total variation of the displacement field as the regulation term. This method is referred to as OFTV. For convenience, the method proposed in this work is referred to as TVDM.

**Figure 3:**
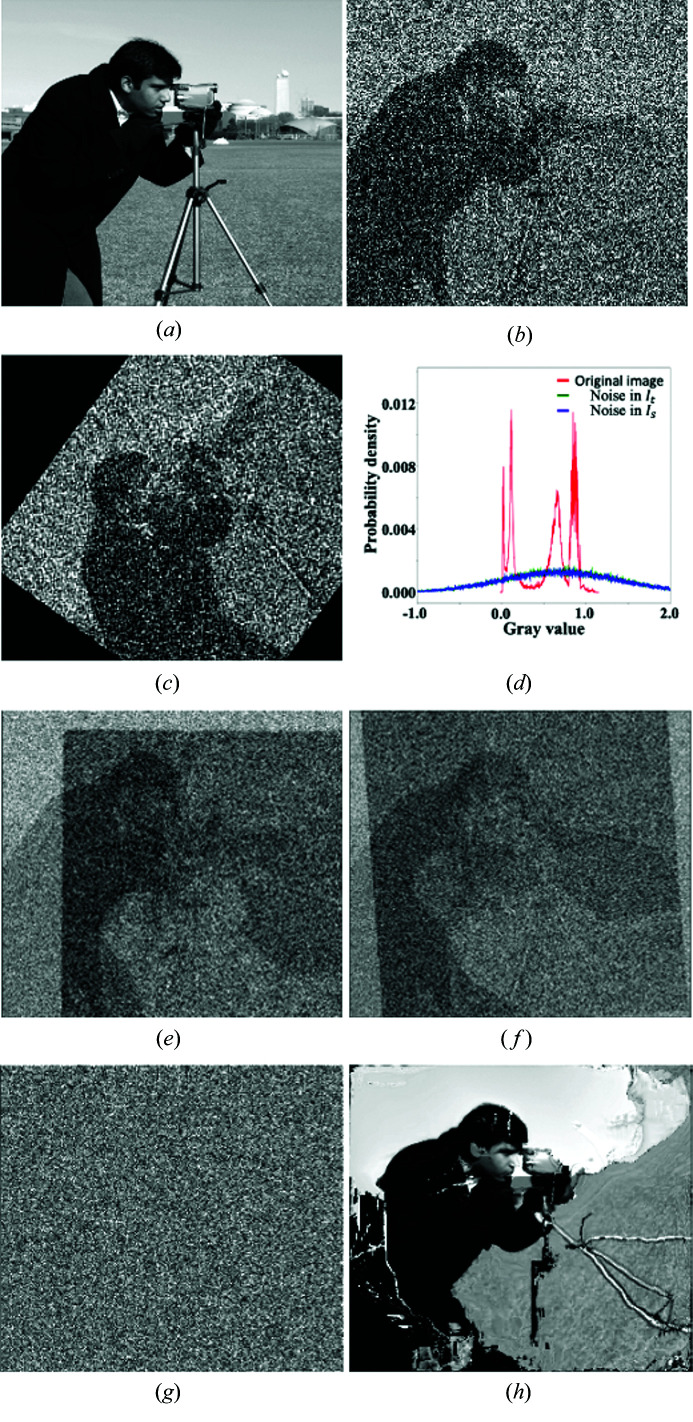
Images used in experiment 1 and results. (*a*) Original image. Panels (*b*) and (*c*) show the synthesized target and source images with additive noises. (*d*) Histograms of (*a*) and additive noises in (*b*) and (*c*). (*e*)–(*g*) Difference images between (*b*) and the registered images with FPC, SSD and TVDM. The edge features in (*e*) and (*f*) indicate that the images are not registered correctly. (*h*) Registered image with OFTV; local distortions in the registered image are obvious.

The first experiment is based on images synthesized from the original noise-free image *I*_0_ in Fig. 3 ▸(*a*). The gray scale of the original image is scaled into the range [0, 1]. The target and source images *I*_t_ and *I*_s_ are synthesized as *I*_t_ = *I*_0_ + *n*_t_ + *b*_t_ and *I*_s_ = *I*_0_ + *n*_s_ + *b*_s_, where *n*_t_ and *n*_s_ are additive noises, *b*_t_ and *b*_s_ are constant background offsets. In this experiment, we choose *n*_t,s_ ≃ 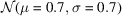 and *b*_t,s_ = 0; 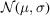 is a noise background following a Gaussian distribution with a mean μ and standard deviation σ. The source image *I*_s_ is translated by **x** = [13.5, 21.3] pixels along the horizontal and vertical directions then rotated by θ = 53.9° relative to the target image *I*_t_. The translation and rotation numbers are chosen randomly. Figs. 3 ▸(*b*) and 3(*c*) show the synthesized target and source images, respectively. Fig. 3 ▸(*d*) shows the gray-value histograms of the noise-free image and the additive noise in Figs. 3 ▸(*b*) and 3(*c*). The noise histogram peak positions being higher than that for the noise-free image suggests that the noise has higher magnitudes than the signals at most image pixels. It is clear that the target and source images in Figs. 3 ▸(*b*) and 3(*c*) lose the details of the weak features in comparison with Fig. 3 ▸(*a*). The registration results given by SSD, FPC and TVDM are [**x**, **θ**] = [17.5, −5.1, −40.2°], [−10.7, −40.5, −35.7°] and [14.2, 20.8, 53.7°], respectively. While the error in the TVDM result is less than 0.7 pixel in the translation and 0.2° in the rotation, the results with SSD and FPC are wildly off. Figs. 3 ▸(*e*)–3(*g*) show the difference maps 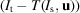 with FPC, SSD and TVDM. Visually, the difference map Fig. 3 ▸(*g*) for TVDM presents no residual edge features but just random noise background. In contrast, Figs. 3 ▸(*e*) and 3(*f*) for FPC and SSD show plenty of residual edge features. Fig. 3 ▸(*h*) presents the transformed source image that is calculated with OFTV based on noise-free target and source images. The image is clearly distorted from the applied local warps. OFTV is designed for non-rigid registration that allows more freedoms for local deformations but enforces fewer constraints to a rigid-body registration even with the TV of the displacement field as the regulation term. Clearly, OFTV is not an effective approach for rigid-body registration tasks. Thus, we will only compare the results with FPC, SSD and TVDM in the following experiments.

More tests are conducted with different noise and backgrounds of various combinations of μ, σ, *b*_t_ and *b*_s_. The results are summarized in Table S1 of the supporting information. All three methods reach results that are in good agreement with the ground truth within ±0.7 pixel for translation shifts and ±0.2° for rotation shift in the relatively low noise cases when μ ≤ 0.4, σ ≤ 0.4 and *b*_t,s_ = 0, with SSD giving slightly better results in this range. FPC becomes instable after μ ≥ 0.5, σ ≥ 0.5 independent of *b*_t,s_ levels. It is noticed that SSD is susceptible to the variant background offsets. Although SSD can reach reasonable results up to μ = 0.5, σ = 0.5 and *b*_t_ = *b*_s_ = 0.6, it fails even with low noise of μ = 0.1, σ = 0.1 when *b*_t_ = 0.6, *b*_s_ = 0.3. In contrast, TVDM provides a solid performance consistently even up to μ = 1.0, σ = 1.0 with either zero or non-zero background offsets in two images.

The performance difference between FPC, SSD and MRTV is not a surprise. As discussed by Tang & Hamarneh (2013 ▸), SSD as a dissimilarity metric does not work well if two images have different background offsets. Cross-correlation-based methods work in such a scenario provided that the background offsets are linearly related. Therefore, the performance of FPC is independent of the constant background offsets in two images. On the other hand, Gaussian noise is unstructured so has uniform distribution in the Fourier space. Filtering in Fourier space will not remove Gaussian noise completely. Thus, FPC becomes unstable when the Gaussian noise level is too high. In the TVDM cases, the TV operation on the difference map of a pair of *I*_t_ and *I*_s_ removes the effect of the constant *b*_t_ − *b*_s_, thus TVDM results are free from the effects of the uniform background offsets in the pair of images. The Gaussian filtering on the target and source images before the difference map operation effectively removes the noise effects in the following TV operation. Therefore, TVDM presents robust performance in this challenging scenario.

**Figure 4:**
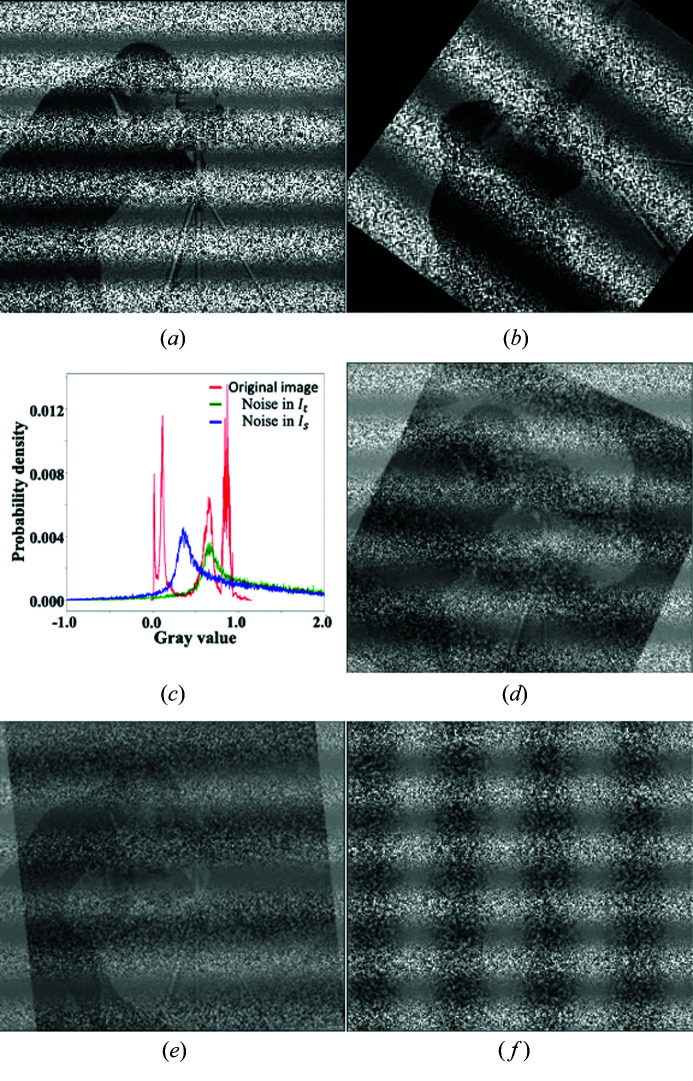
Images used in experiment 2 and results. Panels (*a*) and (*b*) show the synthesized target and source images. (*c*) Histograms of the original image Fig. 3 ▸(*a*) and additive noises in (*a*) and (*b*). (*d*)–(*f*) Difference images between (*a*) and the registered images with FPC, SSD and TVDM.

To further test the performances of different algorithms in the presence of inhomogeneous backgrounds, the target and source images are added to a noise background of the form *b*_t,s_ + sinω_t,s_ *L*_t,s_ *n*_t,s_ in the second experiment, where ω_t,s_ and *L*_t,s_ are the spatial frequencies and spatial variables for the target and source images, respectively. To avoid the correlation between the noise backgrounds, *L*_t_ is set to be the position along the horizontal direction in the target image and *L*_s_ the position along the vertical direction in the source image. The source image is then translated by **x** = [13.5, 21.3] pixels along the horizontal and vertical direction and then rotated by θ = 53.9° relative to the target image. Figs. 4 ▸(*a*) and 4(*b*) show target and source images with backgrounds 0.6 + 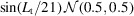 and 0.3 + 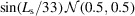 , respectively. Histograms of the original image and the noise backgrounds are presented in Fig. 4 ▸(*c*). The strong and non-uniform noise backgrounds heavily distort the images. Both SSD and FPC fail to register two images as the edge features are still clearly seen in the difference maps between the target and registered source images shown in Figs. 4 ▸(*d*) and 4(*e*). Quantitatively, FPC calculates [**x**, **θ**] = [−25.2, −15.1, 168.7°], and SSD gives [**x**, **θ**] = [17.3, −11.5, −42.1°]. Both are far away from the ground truth [**x**, **θ**] = [13.5, 21.3, 53.9°]. On the contrary, TVDM calculates [**x**, **θ**] = [14.4, 21.2, 53.9°]. As shown in Fig. 4 ▸(*f*), there are no residual edge features visible in the difference map, in contrast to that in Figs. 4 ▸(*d*) and 4 ▸(*e*).

More experiment results under different noise conditions are summarized in Table S2 of the supporting information. It is seen that the level of noise that FPC can handle is reduced to μ = 0.2, σ = 0.2 when sinusoidal modulations are added to the backgrounds. SSD fails to provide reasonable solutions even in the noise-free case because the gray-value levels in the target and source images are impaired due to the different modulations and offset in the backgrounds. TVDM can still provide stable solutions under all the test conditions.

**Figure 5:**
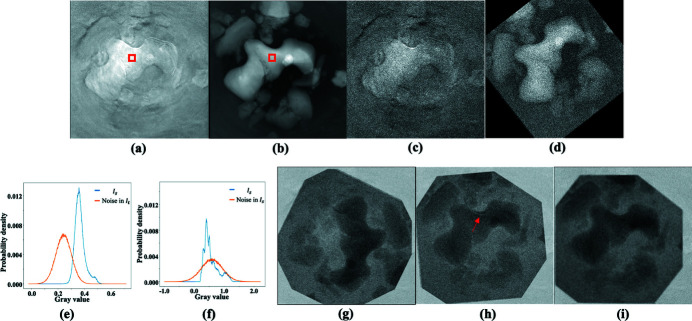
Images used in experiment 3 and results. Panels (*a*) and (*b*) show the original images for the synthesizing target and source images in (*c*) and (*d*), respectively. (*e*) Histograms of (*a*) and the additive noise in (*c*). (*f*) Histograms of (*b*) and the additive noise in (*d*). (*g*)–(*i*) Difference images between (*c*) and the registered images with FPC, SSD and TVDM.

In the third experiment, three algorithms are tested with the images synthesized from real X-ray images. Figs. 5 ▸(*a*) and 5 ▸(*b*) are two X-ray absorption images of a LiCoO_2_ sample taken at 7619 eV and 7729 eV, one below and another above the Co *K*-edge. Two images have had the dark-field background subtracted and normalized with the reference beam images acquired at each energy. In real imaging data, the dominating noise is Poisson noise. However, the average Poisson noise-to-signal ratios in the raw images for Figs. 5 ▸(*a*) and 5 ▸(*b*) are relatively low, 0.13% and 0.17% in the middle regions and 0.45% and 0.54% in the border regions, respectively. The sample has very different absorptions to X-rays at the two X-ray energies, as seen from the different feature contrasts in Figs. 5 ▸(*a*) and 5 ▸(*b*) and the image gray value histograms in Fig. 5 ▸(*e*). Besides, the two images have different backgrounds due to X-ray illumination beam variations at the two energies. Before any following operations, the two images are first manually aligned as the ground truth. To test the algorithms under extreme conditions, Gaussian noise is added to the images again. Fig. 5 ▸(*a*) has added noise of the form 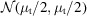 to generate the image in Fig. 5 ▸(*c*), and Fig. 5 ▸(*b*) has added noise of the form 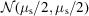 , where μ_t_ = 0.68 and μ_*s*_ = 1.67 are the mean gray values in the regions of interest marked with the red boxes in Figs. 5 ▸(*a*) and 5 ▸(*b*), respectively. The resulting image from Fig. 5 ▸(*b*) is then translated by 37 and −33 pixels along the vertical and horizontal directions and rotated by 38° around its center. The final image is presented in Fig. 5 ▸(*d*). Figs. 5 ▸(*c*) and 5 ▸(*d*) are used as the target and source images in the experiment, respectively. The strong noise backgrounds overwhelm the fine structures in two images. In particular, the weak contrast in the target image is further compromised by the background noise. Fig. 5 ▸(*e*) presents gray value histograms of Fig. 5 ▸(*a*) and the additive noise. Fig. 5 ▸(*f*) presents the gray value histograms of Fig. 5 ▸(*b*) and the additive noise. The histograms of Figs. 5 ▸(*a*) and 5(*b*) have different shapes, and the centers of the noise distributions shift differently from the signal histograms. This indicates that neither the signals nor the additive background noise in Figs. 5 ▸(*c*) and 5 ▸(*d*) are linearly related. Thus, a global scale factor as suggested by Ashburner & Friston (2007 ▸) may not equalize the gray-value levels in Figs. 5 ▸(*c*) and 5(*d*), so the registrations with FPC and SSD may be still affected by the impaired gray levels in the two images (Tang & Hamarneh, 2013 ▸). Nonetheless, a global scale factor, μ_s_/μ_t_, is applied to the target image Fig. 5 ▸(*c*) to roughly equal the gray levels in the two images. The registration results with FPC, SSD and TVDM are [13.2, −7.2, 154.0°], [33.5, −23.7, 33.0°] and [36.9, −31.8, 38.0°]. Compared with the ground truth [**x**, **θ**] = [37, −33, 38°], TVDM gives the closest result. Figs. 5 ▸(*g*)–5(*i*) present difference maps with FPC, SSD and TVDM. Ideally, the residual features in a difference map between the target image and the correctlly aligned source image should just look like Fig. 5 ▸(*c*) since the global scaling on the source image may not perfectly equalize the gray levels of the two images. The incorrect orientation of the features in the middle of Fig. 5 ▸(*g*) indicates that the result with FPC is wrong. The result with SSD is not far from the correct solution, but the double edge feature marked by the red arrow suggests that the result with SSD is also off. In contrast, the difference map with TVDM in Fig. 5 ▸(*i*) shows no visible residual edge features. More experimental results with different levels of additive Gaussian noise are summarized in Table S3 of the supporting information. It is shown that FPC only works in cases when the additive Gaussian noise is at levels μ 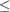 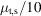 , σ 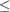 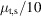 . With higher background noise, FPC results quickly become unstable. SSD fails even to register the original images without additive Gaussian noise due to the quite different backgrounds and features in the image pair. In contrast, TVDM performs well up to the μ = 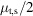 , σ = 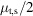 noise level at which the error in the vertical direction translation increases to 1.2 pixel while the errors in the horizontal direction translation and rotation angle are still close to zero.

A typical TXM XANES image dataset is composed of tens to hundreds of images taken at different energy points. One strategy to register a series of images is to use one image as the global target images and register every other image in the series to the target image. The challenge in this approach is that the image contrasts, background features and noise, and gray-value levels may vary dramatically over the entire series. As shown in the second and third examples, FPC and SSD may become instable in such cases. Another strategy is to start with one image as the global anchor and register every two neighbor images progressively from that image. The relative shift of any image from the reference image can be calculated by summing up the shifts of every image pair between these two images. However, even the small registration error in each registration operation may accumulate into a large error at the end. As a trade-off solution to these two strategies, the third strategy is to set one image as the global anchor and split the entire image series into a few small chunks. In each chunk, one image is chosen as the local target image, and every image in the chunk is registered to this local target image. A local target image is registered to its neighbor local target image toward the global anchor image. For instance, if the global anchor image is the number *n*_0_ image in the image series and the chunk size is *n*_c_, then the local target images are […, *n*_0_ − 2*n*_c_, *n*_0_ − *n*_c_, *n*_0_, *n*_0_ + *n*_c_, *n*_0_ + 2*n*_c_,…]. This approach degenerates to the first approach if the chunk size *n*_c_ is set to 1, or to the second approach if the chunk size *n*_c_ is set to the number of images in the entire dataset. Nonetheless, the variations in the images and error accumulation may still pose issues in registering a long image series.

**Figure 6:**
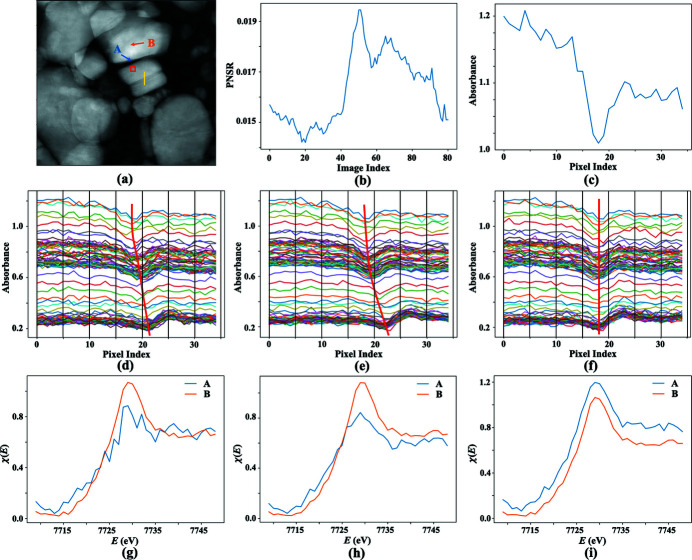
Registration results with a TXM XANES dataset. (*a*) Global reference image. (*b*) Average Poisson noise-to-signal ratio as a function of image index in the region of interest marked with the red box in (*a*). (*c*) Line plot along the line in (*a*). The dip position indicates the crack position in the line. (*d*)–(*f*) Line plots for each image in the dataset after the registrations with FPC, SSD and TVDM, respectively. With an ideal registration, the dip positions in all the lines should be the same. (*g*)–(*i*) XANES spectra at points *A* and *B* marked in (*a*) based on aligned image stacks with FPC, SSD and TVDM, respectively. Alignment errors have more impact on the spectra in the particles’ boundary regions.

In the fourth experiment, three algorithms are tested with real experimental data. The dataset is composed of 101 images of a LiCoO_2_ electrode sample taken at 101 energy points across the Co *K*-edge. The sample was cycled at 0.5 C rate for 100 cycles and measured under *ex situ* conditions. Before registration, each image is normalized by its reference beam image. The third registration strategy with a chunk size of 7 for an image series is utilized in this experiment. Fig. 6 ▸(*a*) shows the global anchor image taken at 7730 eV that is the 51st image in the series. The Poisson noise-to-signal ratio in the small region marked by the red box in Fig. 5 ▸(*a*) is plotted as a function of image index in Fig. 6 ▸(*b*). It is seen that the Poisson noise-to-signal ratio varies in a range between 1.4% and 1.9%. As seen in Fig. 6 ▸(*a*), there is a thin crack across the particle in the middle of the images. A plot along the line across the crack shown in Fig. 6 ▸(*a*) has a sharp dip that indicates the crack position, as shown in Fig. 6 ▸(*c*). Ideally, the line plots for each image should have the dip at the same position if all the images are correctly aligned. Figs. 6 ▸(*d*) and 6 ▸(*e*) are line plots based on the registration results with FPC and SSD. Clearly, the line plot dip positions drift between different images. The drift range is between [−2, +1.5] pixels for FPC and [−2, +2.5] pixels for SSD. On the contrary, the line plots for TVDM in Fig. 6 ▸(*f*) show that the dip positions stay tightly within a ±1 pixel range over 101 images that have significant variance in the gray-value levels.

Precise alignment of the entire XANES image stack is crucial for obtaining an accurate valence state distribution of the concerned elements, especially in the particles’ boundary regions. Figs. 6 ▸(*g*)–6(*i*) plot the XANES spectra at the points *A* and *B* marked in Fig. 6 ▸(*a*), based on the FPC, SSD and TVDM registration results. Point *A* is on the boundary, and point *B* is in the middle, of a LiCoO_2_ particle. While the spectra for point *B* show no fundamental differences in Figs. 6 ▸(*g*)–6(*i*), the spectra in Figs. 6 ▸(*g*) and 6 ▸(*h*) for the point *A* obtained from the aligned image stacks with FPC and SSD present significant distortions due to the alignment errors. In comparison, the spectrum for point *A* obtained from the aligned image stack with TVDM is smooth and similar to that at point *B*. This is an expected result since LiCoO_2_ single crystals tend to have a uniform state of charge under slow charging conditions (Xu *et al.*, 2017 ▸).

## 5. Discussions

The proposed TVDM method demonstrates superior performance over the other two intensity-based conventional rigid registration methods in three experiments. The effects of the TV operation in TVDM are different from those in the conventional TV regulation on the displacement field in deformable registration methods. As shown in the first experiment, the TV regulation in the optical flow method does not provide a sufficient rigid-body transform constraint in the rigid registration cases. In TVDM, the Gaussian filter together with TV operation on the DM of the image pair can effectively reduce various slow background variations while keeping the edge features. This is different from band filters such as the one implemented in the FPC method that cannot remove noise in the reserved bandwidth. In all three experiments, TVDM works well under all test conditions whilst FPC only works in relatively low-noise cases.

TVDM still requires gray value scaling if two images have very different gray-value levels. However, it is not sensitive to the accuracy of the global scaling factor. As shown in the third experiment, it works even when the gray value histograms of two images are not completely overlapping. In contrast, SSD is sensitive to the scaling factor choice in such a case. The output may change if the scaling factor is varied. That makes the SSD method still subjective to visual evaluations and manually tweaking of its parameters according to the image conditions.

The parameters for TVDM are not sensitive to the application conditions. TVDM has few arguments for adjusting its performance. The default values of most arguments work in most cases. The parameters in the translation offset search are the number of resolution levels and the search range in each level. These two parameters determine the overall search range. The default setting is 5 for the number of resolution level and 8 for the search range at each level, which provides an overall search range of [−64, 64] pixels in each dimension. The rotation angle search uses the differential evolution method whose key arguments are search range, number of population (NP), mutation factor (F) and the crossover factor (CR) (Storn & Price, 1997 ▸). In the DE implementation in *Scipy.Optimize*, the default setting is 0.7 for CR and randomly dithering in the range [0.5, 1) on a generation-by-generation basis for F. These default settings are inherited in TVDM. To avoid local minima in angle search, the default setting for NP is set to 100 to ensure sufficient sampling in the search space. The default angle search range is set to [−10°, 10°]. This assumes a rough estimation on the relative rotation between two images that is not difficult to do. The most important parameter in TVDM is the Gaussian filter kernel width. The Gaussian filter is used to reduce the noise outlier presence in the DM of two images. Its value balances the noise removal and the edge preservation. A large kernel width can smear the edge features in the images that may reduce the registration accuracy. In practice, a kernel width around 1 is found to be sufficient in most cases, thus the default value for the Gaussian filter kernel width is set to 1. These default argument values are used in all three experiments. The success of TVDM in all the test cases shows the robustness of the proposed method in rigid registration applications.

Although it is not tested, the results of the third experiment suggest that the proposed TVDM method is expected to work in the multimodal image rigid registration tasks if two images share common feature boundaries but have varying contrasts between different features.

## 6. Summary

A new dissimilarity metric based on the total variation of the difference map between an image pair is proposed for rigid registration applications. A rigid registration method based on a new metric, TVDM, is tested and compared with the Fourier analysis-based phase correlation and sum of squared difference metrics. The results demonstrate the validity and robustness of TVDM. TVDM overperforms the other two intensity-based rigid registration methods, one based on Fourier phase correlation and the other based on the sum of squared difference, under both simulated and real experimental conditions. The proposed method is expected to find broad applications in image registration tasks, *e.g.* imaging-based nano-XANES data analysis, in which varying levels of noise and backgrounds could be pronounced. TVDM has been integrated into *TXM_Sandbox* (Xiao *et al.*, 2022 ▸) that will be released at https://github.com/xianghuix/TXM_Sandbox.

## Supplementary Material

Supplementary Information in which the results for experiments 1, 2, and 3 under extensive test conditions are included. DOI: 10.1107/S1600577522005598/mo5258sup1.docx
