# Supplementary material for: Rigid registration algorithm based on the minimization of the total variation of the difference map
Source: J Synchrotron Radiat. 2022 Jun 20;29(Pt 4):1085–94. doi: 10.1107/S1600577522005598 (PMC9255568; doi:10.1107/S1600577522005598)
Supplement: Supplementary file 1 [file s-29-01085-sup1.docx]

1. Symmetries that break the uniqueness in translation and angle searches

When the feature patterns, no matter point based or intensity based, pose certain symmetries, both translation and angle searches for matching two images may not return unique solution. For instance, for the target and source images shown in Supplementary Figs. 1(a) and (b), it won’t be possible to find a unique angle to match the cycles in the images. In fact, if the feature pattern in the pair of images pose central symmetry around some rotation center, there won’t be possible to find a unique rotation angle to match two images.


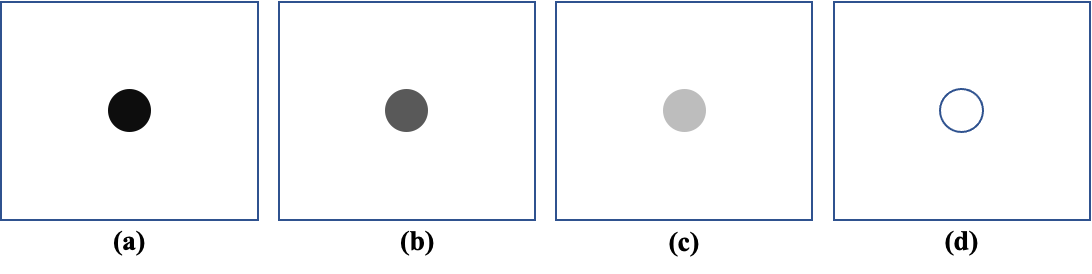


Supplementary Figure 1. (a) and (b) are the target and source images, respectively. (c) is the differential map between (a) and (b). (d) gradient of (c). Apparently, either (b) is rotated 0° or 180° gives the same differential map (c) and the gradient map (d). Therefore, neither TVDM nor SSD can find a unique angle to match (a) and (b).

In a case like that in Supplementary Fig. 2, in which 2(a) is the target image and 2(b) and 2(e) are the source images with two different translation offsets. The target and source images have rotation offsets. Supplementary Figure 2(c) is the difference map between 2(a) and 2(b), and 2(f) is the difference map between 2(a) and 2(e). Supplementary Figure 2(d) is the gradient of 2(c), and 2(g) is the gradient of 2(f). It is obvious that the sums of squared difference based on Supplementary Figures. 2(c) and 2(f) are same. Similarly, the *L1-*norm on 2(d) and 2(g), which are the total variations of the difference maps 2(c) and 2(f), are same. In this case, neither the sum of squared difference nor the total variation of the difference can discriminate Supplementary Figures. 2(b) and 2(e) that have two different translation offsets.


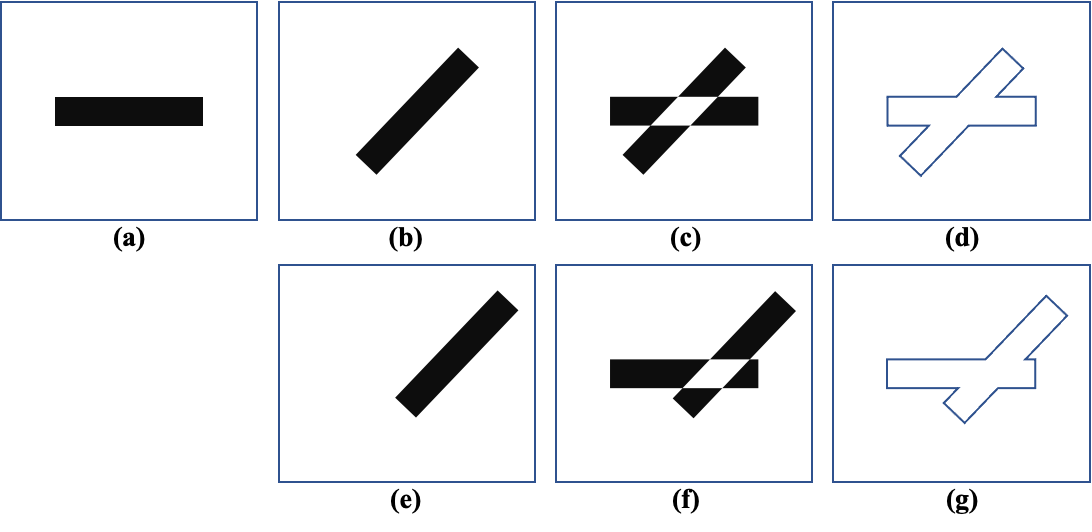


Supplementary Figure 2. (a) and (b) are the target and source images. (c) the difference map between (a) and (b), and (d) the gradient of (c). (e) another source image with a different translation offset from (a). (f) the difference map between (a) and (e); (g) the gradient of (f). It is obvious that the integrals of (c) and (f) and the integrals of (d) and (g) are same, respectively.

1. Experiment 1

Two independent sets of noise in the form $b_{i}\mathcal{+N}\left( \mu, \sigma\right), i=t, s$ are added to the original image shown in Fig. 3(a) to generate target image $I_{t}$ and source image $I_{s}$. The noise condition is referred in the form $(\mu, \sigma, b_{t}, b_{s})$. To generate the source image, the noise image is translated by 13.5 and 21.3 pixels along horizontal and vertical direction then rotate by $53.9^{\circ}$. The test result is expressed as $(t_{x}, t_{y}, \theta)$, where $t_{x}$ and $t_{y}$ are the translation offsets along horizontal and vertical directions, and $\theta$ the rotation offset. TABLE 1 summarizes the results from experiment 1. The default band filter setting in PC is used in the tests. The intensity scaling between two images is manually tweaked to get the best results shown in the table.

TABLE 1. SUMMARY OF EXPERIMEENT 1

| $(\mu, \sigma, b_{t}, b_{s})$ | FPC | SSD | TVDM |
| --- | --- | --- | --- |
| (0, 0, 0, 0) | (14.2, 21.1, 53.9) | (13.5, 21.3, 53.9) | (14.1, 21.1, 53.9) |
| (0.1, 0.1, 0, 0) | (14.1, 21.1, 54.0) | (13.5, 21.3, 53.9) | (14.2, 21.1, 53.9) |
| (0.2, 0.2, 0, 0) | (14.2, 21.1, 53.7) | (13.5, 21.3, 53.9) | (14.1, 21.1, 53.9) |
| (0.3, 0.3, 0, 0) | (14.0, 21.2, 54.0) | (13.5, 21.4, 53.9) | (14.1, 21.2, 53.9) |
| (0.4, 0.4, 0, 0) | (14.7, 21.1, 53.6) | (13.4, 21.4, 54.0) | (14.2, 21.2, 53.9) |
| (0.5, 0.5, 0, 0) | (1.6, -3.5, 5.0) | (13.2, 21.4, 54.1) | (14.0, 21.0, 54.0) |
| (0.6, 0.6, 0, 0) | (-42.2, 11.9, 144.0) | (32.3, 20.3, -3.8) | (14.1, 21.2, 53.9) |
| (0.7, 0.7, 0, 0) | (-10.7, -40.5, -35.7) | (17.5, -5.1, -40.2) | (14.2, 20.8, 53.7) |
| (0.8, 0.8, 0, 0) | (-26.7, 29.2, -179.7) | (12.8, -15.6, -41.6) | (14.0, 20.9, 54.2) |
| (0.9, 0.9, 0, 0) | (-56.4, -4.3, 12.0) | (12.8, -17.8, -41.9) | (14.2, 21.0, 53.7) |
| (1.0, 1.0, 0, 0) | (-20.5, 21.9, 3.7) | (16.4, -7.4, -40.9) | (13.9, 20.9, 53.8) |
| (0.1, 0.1, 0.6, 0.3) | (14.1, 21.0, 53.9) | (14.5, -28.5, -36.2) | (14.1, 21.0, 53.9) |
| (0.2, 0.2, 0.6, 0.3) | (14.0, 21.1, 53.8) | (14.4, -28.7, -36.2) | (14.0, 21.0, 53.9) |
| (0.3, 0.3, 0.6, 0.3) | (14.1, 21.1, 53.9) | (14.1, -29.1, -36.1) | (14.1, 21.0, 53.9) |
| (0.4, 0.4, 0.6, 0.3) | (14.1, 21.0, 53.9) | (13.9, -29.1, -36.3) | (14.1, 21.1, 53.9) |
| (0.5, 0.5, 0.6, 0.3) | (14.2, 21.1, 54.2) | (14.2, -28.7, -36.3) | (14.4, 21.1, 53.8) |
| (0.6, 0.6, 0.6, 0.3) | (27.4, 36.6, -23.7) | (13.7, -28.9, -36.2) | (14.0, 21.2, 53.9) |
| (0.7, 0.7, 0.6, 0.3) | (93.4, -21.2, 2.0) | (13.6, -27.1, -36.7) | (14.1, 21.1, 54.1) |
| (0.8, 0.8, 0.6, 0.3) | (3.6, -2.3, 3.3) | (14.1, -27.6, -37.4) | (14.1, 21.1, 54.0) |
| (0.9, 0.9, 0.6, 0.3) | (-6.7, -31.3, -172.2) | (18.1, -16.9, -40.5) | (14.0, 21.2, 53.8) |
| (1.0, 1.0, 0.6, 0.3) | (-14.0, -46.0, -171.9) | (13.4, -20.2, -36.7) | (14.2, 21.1, 53.8) |

1. Experiment 2

The procedure to generate target and source images is same as that in Experiment 1 except the form of noise is different. The translation and rotation values in this experiment are same as that in Experiment 1. The noise is in the form $b_{i}+\sin\omega_{i}L_{i}\mathcal{\cdot N}\left( \mu, \sigma\right), i=t, s$, where $\omega$ is the background modulation frequency and $L$ is the spatial variable. In this experiment, the modulation frequency is fixed to 21 for the target image and 33 for the source image. For the target image, the modulation direction is along vertical direction. For the source image, the modulation direction is along horizontal direction before the image is rotated. The noise condition and the registration result are expressed in the same forms as that in Experiment 1. The default band filter setting in PC is used in the tests. The intensity scaling between two images is manually tweaked to get the best results shown in the table.

TABLE 2. SUMMARY OF EXPERIMENT 2

| ($\mu,\sigma,b_{t}, b_{s}$) | FPC | SSD | TVDM |
| --- | --- | --- | --- |
| (0, 0, 0.6, 0.3) | (14.2, 21.1, 53.9) | (13.8, -29.1, -36.2) | (14.1, 21.1, 53.9) |
| (0.1, 0.1, 0.6, 0.3) | (14.1, 21.1, 53.9) | (13.6, -29.1, -36.2) | (14.1, 21.1, 53.9) |
| (0.2, 0.2, 0.6, 0.3) | (14.0, 21.1, 54.1) | (17.5, -10.4, -42.0) | (14.2, 21.1, 53.9) |
| (0.3, 0.3, 0.6, 0.3) | (-11.1, 27.3, 21.8) | (16.9, -9.7, -42.3) | (14.3, 21.1, 53.8) |
| (0.4, 0.4, 0.6, 0.3) | (68.2, -77.8, -36.9) | (16.5, -12.6, -42.8) | (14.4, 21.0, 54.0) |
| (0.5, 0.5, 0.6, 0.3) | (-25.2, -15.1, 168.7) | (17.3, -11.5, -42.1) | (14.4, 21.2, 53.9) |

1. Experiment 3

Two X-ray images of a LiCoO_2_ particle sample taken at two different X-ray energies are used to synthesize the test images. The noises in the form $\mathcal{N}\left( m_{\mu}\mu_{t,s}, m_{\sigma}\sigma_{t,s} \right)$ are added into two images independently, where $\mu_{t}=\sigma_{t}=0.68$, and $\mu_{s}=\sigma_{s}=1.67$; $\mu_{t}$ and $\mu_{s}$ are calculated as the mean in the regions of interest marked in Fig. 5(a) and 5(b). The source image shown in Fig. 5(b) is translated by 37 and -33 pixels along horizontal and vertical direction then rotate by $38^{\circ}$. In TABLE 3, the registration result is expressed as $(t_{x}, t_{y}, \theta)$, where $t_{x}$ and $t_{y}$ are the translation offsets along horizontal and vertical directions, and $\theta$ the rotation offset. The default band filter setting in the chosen FPC is used in this experiment. The intensity scaling between two images is manually tweaked to get the best results shown in the table.

TABLE 3 SUMMARY OF EXPERIMENT 3

| ($m_{\mu}, m_{\sigma}$) | FPC | SSD | TVDM |
| --- | --- | --- | --- |
| (0, 0) | (37.7, -33.0, 37.9) | (33.1, -26.0, 33.2) | (37.4, -32.7, 37.8) |
| (0.1, 0.1) | (37.7, -32.9, 37.9) | (34.5, -26.4, 34.1) | (37.3, -32.4, 37.8) |
| (0.2, 0.2) | (29.8, -14.0, -21.7) | (34.0, -25.7, 33.2) | (37.3, -32.9, 37.6) |
| (0.3, 0.3) | (2.1, -0.4, -172.5) | (6.1, 2.2, 16.8) | (37.1, -32.7, 37.4) |
| (0.4, 0.4) | (-65.3, 5.5, -163.6) | (0.2, 11.2, 12.5) | (36.6, -33.0, 37.2) |
| (0.5, 0.5) | (13.2, -7.2, 154.0) | (33.5, -23.7, 33.0) | (36.9, -31.8, 38.0) |
